# Automatic gait evoking in healthy adults through Vojta’s peripheric somatosensory stimulation: a double-blind randomized controlled trial

**DOI:** 10.1186/s12984-024-01470-2

**Published:** 2024-10-01

**Authors:** Luis Perales-López, Ismael Sanz-Esteban, Camen Jiménez-Antona, J. Ignacio Serrano, Ana San-Martín-Gómez, Xisca Vives-Gelabert, Roberto Cano-de-la-Cuerda

**Affiliations:** 1 Department of Neurorehabilitation. Numen Foundation, Madrid, Spain; 2 Department of Physiotherapy. Physical Therapy and Health Research Group, Faculty of Sport Sciences, Universidad Europea de Madrid, Madrid, Spain; 3 Department of Physical Therapy, Occupational Therapy, Rehabilitation and Physical Medicine, Faculty of Health Sciences, Universidad Rey Juan Carlos (URJC), Alcorcón, Madrid Spain; 4 Neural and Cognitive Engineering Group (gNeC), Center for Automation and Robotics CSIC- UPM (CAR CSIC-UPM), Madrid, Spain

**Keywords:** Automatic gait, Kinematics, Kinetics, Vojta therapy, Reflex locomotion

## Abstract

**Background:**

To study the effects of different interventions on automatic gait processing in contrast with voluntary gait processing in healthy subjects.

**Methods:**

A double-blind randomised controlled trial was designed (120 able-body persons between 18 and 65 years old entered and completed the study), with pre-intervention and post-intervention assessments using the 6-Minute Walk Test (6MWT). The participants were randomly distributed into four groups. Prior to intervention, all participants performed voluntary gait on the ground (VoG) in a calibrated circuit following the 6MWT. The presence of automatic gait (AG) was explored post-intervention without a voluntary demand in the same circuit following the 6MWT. Each group received a different intervention for 30 min: Vojta stimulation, MOTOMED^®^ at no less than 60 revolutions/minute, treadmill walking at 3 km/h, and resting in a chair (control). The main assessment, conducted by a blinded rater, was the difference in distance covered (in meters) during the 6MWT between pre- and post-intervention. Surface electromyography (sEMG) average root mean square (RMS) signals in the right tibialis anterior, right soleus, right rectus femoris, and right biceps femoris were also considered outcome measures.

**Results:**

The Vojta group was the only one that initiated AG after the intervention (476.4 m ± 57.1 in VoG versus 9.0 m ± 8.9 in AG, *p* < 0.001) with comparable kinematics and EMG parameters during voluntary gait, except for ankle dorsal flexion. Within the Vojta group, high variability in kinematics, sEMG activity, and distance covered was observed.

**Conclusions:**

AG isolation is approachable through Vojta at only one session measurable with the 6MWT without any voluntary gait demand. No automatic gait effects were observed post-intervention in the other groups.

**Trial registration:**

NCT04689841 (ClinicalTrials.gov).

## Background

Studies on gait in able-bodied persons have served to identify common kinematic and kinetic patterns related to locomotion and to identify deviations from normality [1, 2]. In clinical practice, observational scales are the most common approach used to assess gait patterns, but instrumental systems provide more objective data on kinematic and kinetic parameters. Nevertheless, instrumental systems require custom instrumentation, they take a longer time, and their use in clinical practice is not always available [3].

Takakussaki described three levels of neural processing in gait: voluntary (VoG), emotional (EG) and automatic (AG) [4]. AG processing related to gait in humans has been shown to be localized in the mesencephalic locomotor region (MLR), in addition to other areas, such as the basal ganglia and cerebellum [5]. Therefore, although gait parameters may be influenced by the cortex depending on the goals or needs of the tasks, they do not seem to be indispensable for controlling posture and movement during walking [6]. In this context, the study of AG without an external trigger has been conducted in a very limited number of studies [4, 5].

Vojta reflex locomotion therapy (RLT) is considered a bottom-up rehabilitation approach [6], and it is based on the global activation of innate locomotion patterns evoked by sustained pressure stimulation on specific body points from a specific initial position [7]. Involuntary postural and motor responses may be activated by RLT [8], and their effects have been studied at several age ranges [9], and in different neurological disorders [10–12]. However, its neurophysiological justification for AG has not been demonstrated in able-bodied individuals.

In this context, most rehabilitation techniques seek to generate VoG in people with neurological disorders, considering the difficulties in differentiating between EG and AG neural processing. However, in situations of functional limitation, when there is a voluntary inability to initiate or execute movement, rehabilitation approaches that increase AG activation might be justified. In this sense, Malone et al. [13] reported that for gait rehabilitation, nonconscious training without verbal commands about the walking process provides greater benefits for learning to walk in an automatic manner. Consequently, studies justifying such hypotheses would be relevant and should be conducted in able-bodied persons to justify its potential use in people with neurological pathology at a later stage.

Therefore, the aim of this research was to study the effects of RTL, compared with those of different interventions and a control group, on AG in able-bodied persons. Our initial hypothesis was that, compared with other interventions, the AG pattern could be triggered with a single session of RTL because, with the RLT, the dissociation between the AG and the VoG and/or EG can be elicited by suppressing self-initiated gait commands.

## Methods

### Study design

A double-blind randomised controlled trial (RCT) was designed with a pre-test and a post-test assessment (ClinicalTrials: NCT04689841). The Consolidated Standards of Reporting Trials (CONSORT) statement was consulted to help authors improve the reporting of the RCT. A non-probabilistic sampling of consecutive cases was performed. The effect size estimated for the main measure (time spent walking the first 3 m) was 0.30, considering a statistical power of 0.95 and an alpha error of 0.05 for the ANOVA tests of the four groups according to the G.power software (version 3.1.9). Accordingly, a minimum of 102 participants were needed. Accounting for a 10% potential loss, a sample size of 120 participants (30 per group) was considered for this study.

**Fig. 1:**
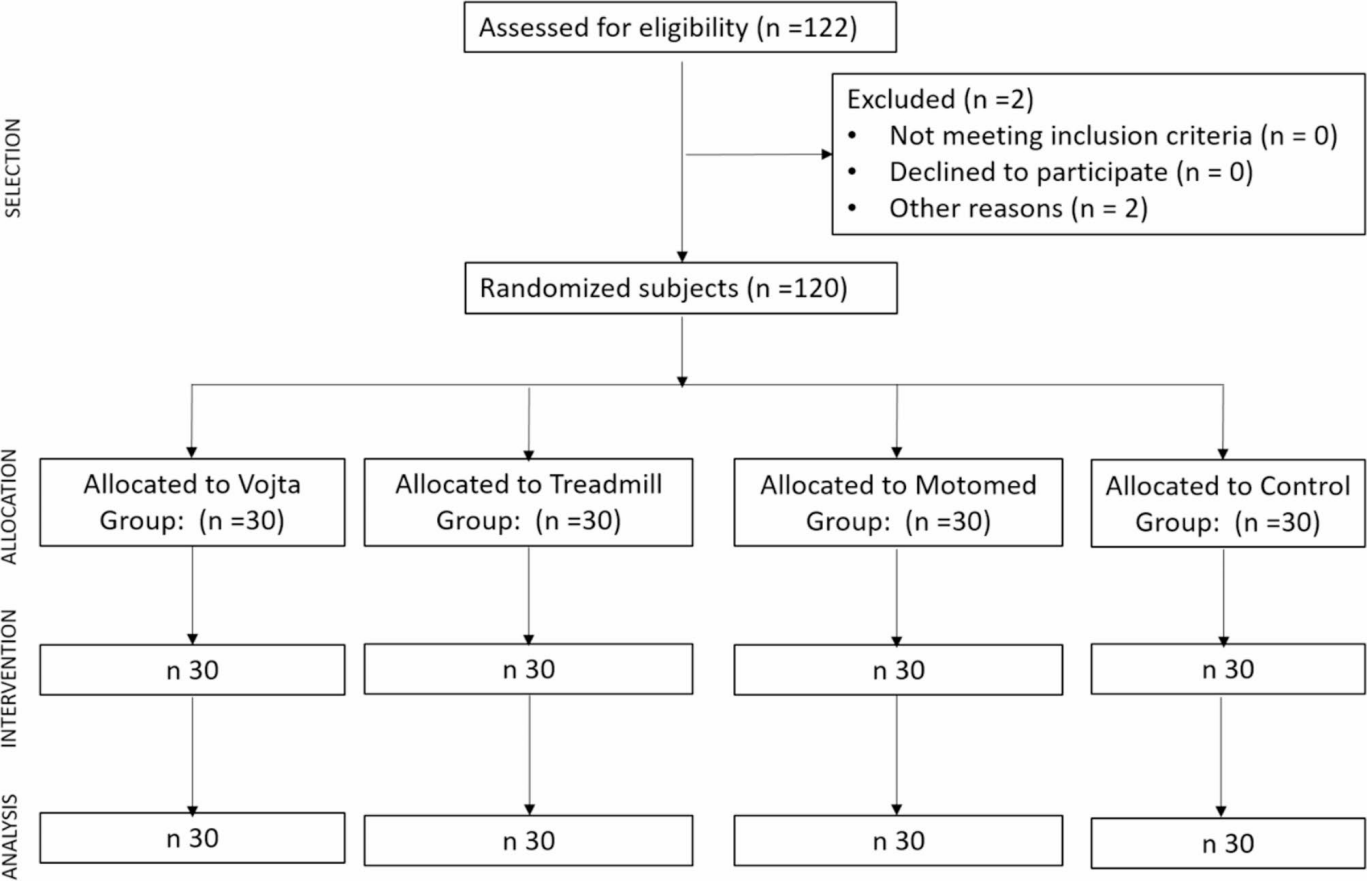
CONSORT flow diagram

The sample was recruited from the Universidad Europea de Madrid (Madrid, Spain). All able-bodied individuals who met the inclusion criteria were randomized into four study groups by a blind investigator —the Vojta group, the treadmill group, the Motomed group or the control group—using the QuickCalcs GraphPad^®^ software with a computer-generated sequence.

Allocation was performed by a blinded investigator of any intervention and evaluations performed. Figure 1 shows the CONSORT flow diagram.

### Participants

The inclusion criteria were as follows: aged between 18 and 65 years; no neurological, cardiorespiratory, or systemic disease; able to complete the 10-meter walk test (10MWT) at a comfortable speed of 15 s; and able to perform a deep squat without assistance. The exclusion criteria were having a prosthesis in any extremity, not having undergone surgery in the last 3 months, pregnancy, having arthrodesis in any extremity, ankylosis or bone fixations in any extremity, soft tissue pathologies or range of movement limitations in the lower limbs.

All the subjects included in the present study were informed of the objectives, protocol and possible risks. All the participants voluntarily agreed to participate and provided their consent in writing. The protocol and informed consent provided to all the subjects were approved by the Research Ethics Committee of Rey Juan Carlos University (reference: 2404201908919). This randomised controlled trial was registered at ClinicalTrials.gov (Identifier: NCT04689841).

### Interventions

**Fig. 2:**
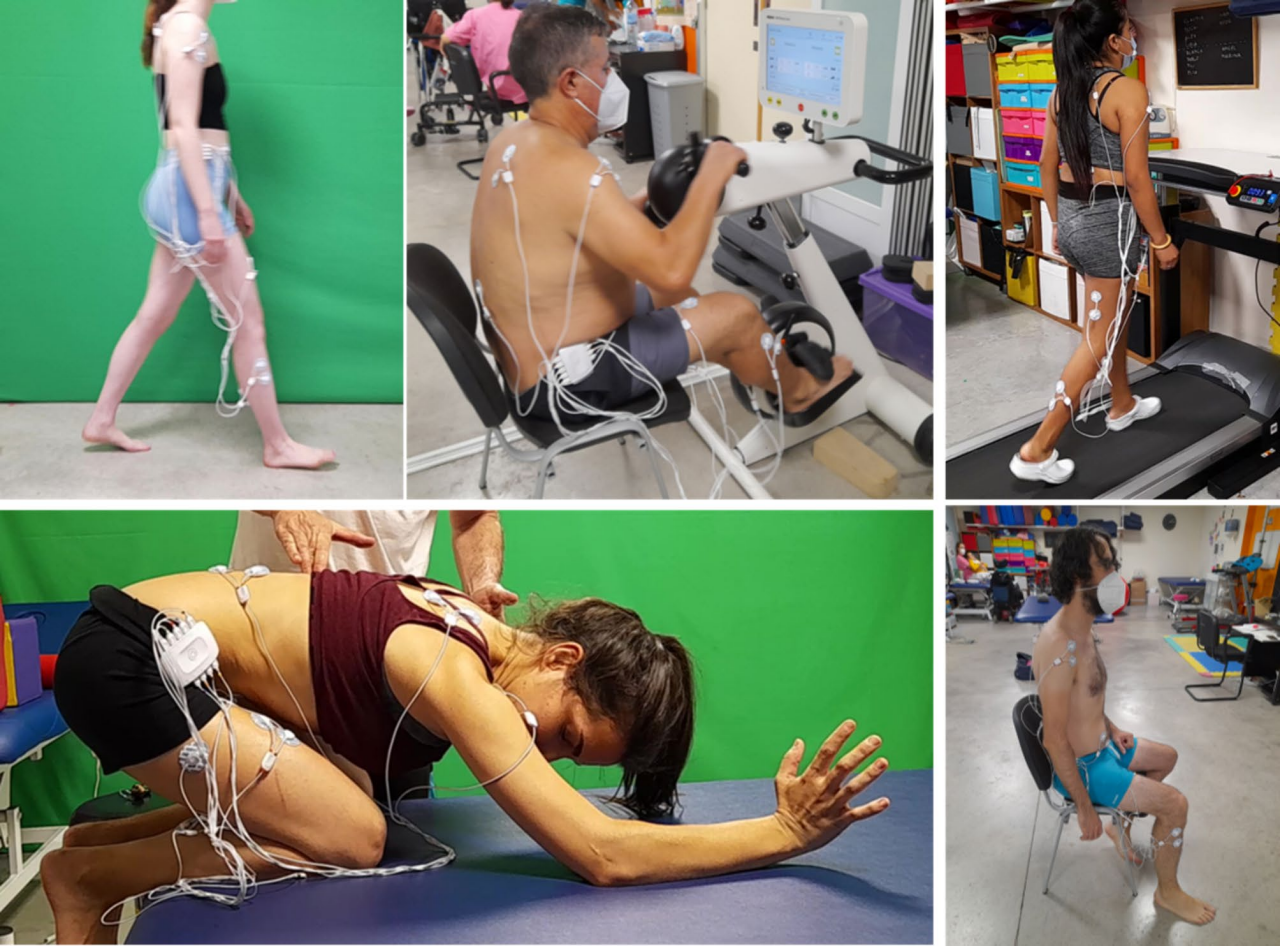
Interventions and voluntary gait pre-intervention (6MWT). Top left: voluntary gait on the floor (6MWT pre-intervention). Top-center: Motomed group (MG); Top-right: Treadmill group (TG). Bottom left: Vojta Group (VG) initial position (maximal flexion on the legs, facial arm: 135° arm flexion, 40° elbow flexion and the nuchal arm extending along the body). Bottom-right: Control group (CG)

The participants were randomly distributed into one of the 4 intervention groups established (Fig. 1). Prior to group assignment, all the participants performed a voluntary gait on the ground (VoG) in a calibrated circuit following the 6MWT instructions [14] (Fig. 2 top-left).

#### Vojta group (VG)

RLT was performed with a defined starting position (Fig. 2 bottom-left). The posture was changed reciprocally for a maximum of 7 min per side, twice on each side. The stimulation areas were located at the external protuberance of the calcaneus and gluteus area [6, 7]. Other areas, such as the trunk area and acromion, were stimulated, and the head and sacrum were guided resistance areas following the RLT technique [8]. Intervention was performed by a Physical Therapist specialized in the application of Vojta therapy with adults.

#### Motomed® group (MG)

Motomed^®^ Group (MG) muvi de Rech, a device for simultaneous and coordinated pedaling of legs and arms with servo-assistance, was used [15]. Active pedaling mode was programmed at a rate of no less than 60 revolutions per minute (rpm) for 30 min (Fig. 2 top center).

#### Treadmill group (TG)

Walking was carried out on a treadmill brand BH model LK700WS with a walking surface of 550 × 1350 mm at a speed of 3 km/h for 30 min [16]. No additional weight suspension system was used, nor was the inclination gradient active on the treadmill (Fig. 2 top-right).

#### Control group (CG)

The participants included in this group did not receive any kind of intervention and were asked to sit still in a chair for 30 min (Fig. 2 bottom-right).

### Outcome measures

All outcome measures were measured by two blinded investigators with respect to the interventions carried out.

#### 6-minute walking test pre-intervention (voluntary)

The 6MWT was performed on all participants following international recommendations. The subjects stood with their right foot forward and their weight resting on the left foot with a normal passage width, and voluntary walking started at his normal speed on the floor on a calibrated circuit [14]. We evaluated the distance covered, and we also extracted and evaluated the percentage of the step cycle executed, considering a single complete step as 100%. In addition, the time spent walking the first 3 m was also extracted and considered.

#### 6-minute walking reflex test post-intervention (nonvoluntary)

For the pre-intervention 6MWT, the participants started in the same starting position. The subject remained in this position following the command “STOP”, which was reinforced with a second command: “DO NOT MAKE ANY VOLUNTARY MOVEMENT”. After such instructions were given to all the subjects, the post-intervention 6MWT assessment began to assess the presence of AG. We evaluated the distance covered in meters and the percentage of the step cycle executed, considering a complete step as 100% of the cycle of an automatic walk (non-voluntary), in case this activation occurred in the post-intervention evaluation. The gait cycle is considered the sequence of movements that occur between two successive contacts of the same foot with the ground [3, 10]. The cutoff point to consider the presence of AG was established at 20% of the gait cycle in this study. This percentage corresponds to the initial single support stance phase.

#### Surface electromyography (sEMG)

Measurements at the first 3 m of the 6MWT pre- and post-intervention (with a nonwalking command) were recorded. The distance was considered, and it was captured by the video camera in the sagittal plane for subsequent kinematic analysis with Kinovea^®^ software.

A Biosignalplux^®^ device for sEMG recording was used. The signal was amplified and digitized by a Biosignalsplux Hub 8-channel device (Plux Wireless Biosignals S.A., Lisbon, Portugal), transmitted and stored with OpenSignal software. All the measurements were taken only on one side (right), and 100% gait cycle data were obtained in each complete cycle on the measured side. Because able-bodied persons were recruited and able to walk (and both lower limbs would move through all phases of the cycle with practically the same activity), obtaining EMG data from both sides was not considered relevant since it was not intended to compare the activity between paired muscles. In addition, as the right side was in the foreground (closer to the rater camera), this side was completely visible in the sagittal plane for posterior analysis.

Surface ElectroMyoGraphy for the Non-Invasive Assessment of Muscles (SENIAM) [17] indications were followed to place the eight electrodes: the tibialis anterior (TA), soleus (SO), rectus femoris (RF), biceps femoris (BF), external abdominal oblique (EO), paraspinal (PV), mid trapezius (MT) and anterior deltoid (DA) electrodes.

#### sEMG data analysis

**Fig. 3:**
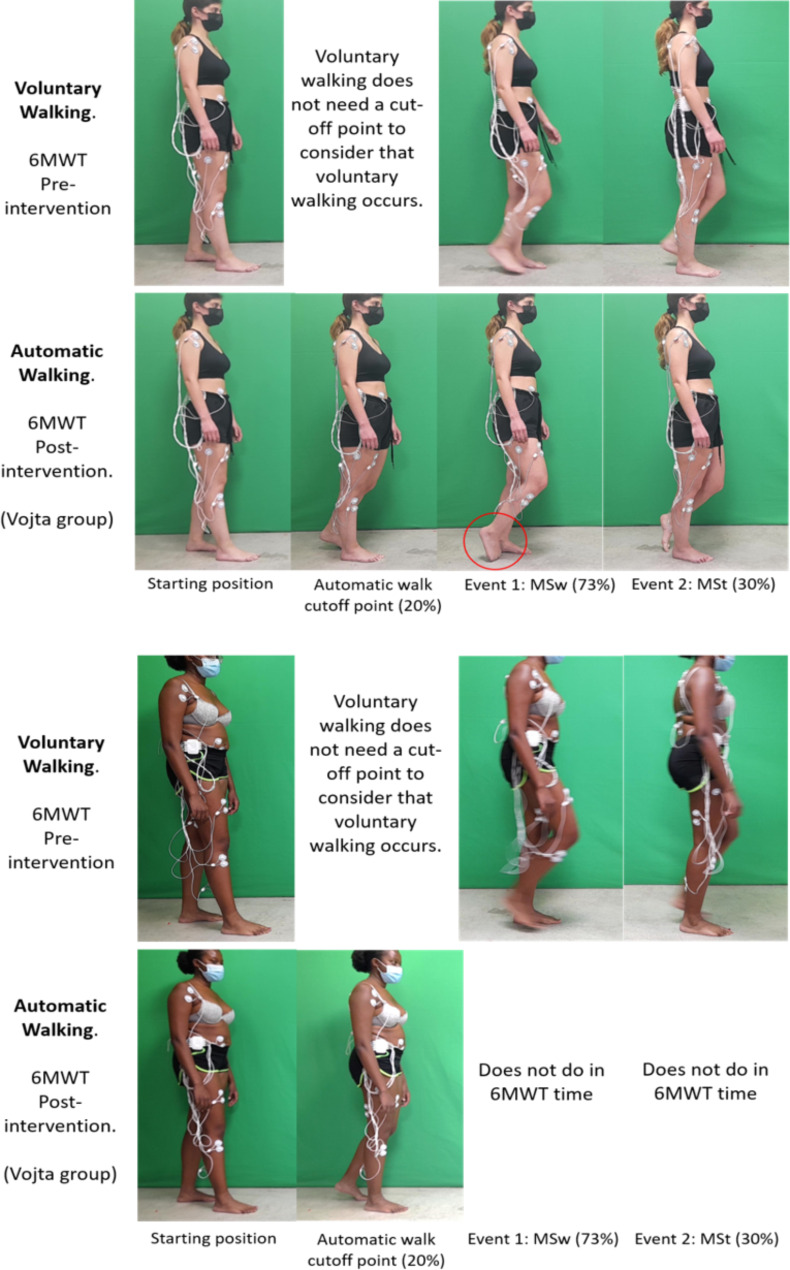
Initial position, EMG cutoff events for gait phase determination, and kinematic analysis. Top: Vojta Group (VG) example with at least a complete cycle of voluntary gait at the 6MWT. At event-1 in VoG, a dorsal flexion deficit is observed (red circle); bottom: VG case example in which VoG is initiated without completing a gait cycle

Intergroup comparisons were performed. The AG threshold initiated in the 6MWT post-intervention was considered for all groups as a 20% gait cycle. Two gait cycle reciprocal events were selected for EMG contrast during the 6MWT before and after intervention. The first event corresponds to the right leg’s initial swing phase (IS), and the second event corresponds to the right mid-stance phase (MS) (Fig. 3).

EMG was captured at 1000 Hz and then processed in Matlab v2017b (The MathWorks Inc., Natick, Massachusetts, USA) according to standard procedure [18]. Initially, the first gait cycle was segmented and extracted from the total recording according to the manually annotated events. Then, the signal in that segment was subsequently detrended by subtracting any offset or linear trend. After that, a zero-phase 6th-order Butterworth digital filter was applied between 10 Hz and 495 Hz. The segment was then split into the two consecutive gait phases considered. Finally, for each gait phase segment, the envelope of the signal was extracted by computing the root mean square (RMS) in sliding windows of 150 ms (150 samples) and then normalized (division) to the maximum RMS value in each segment. Each segment was then quantified by the average normalized RMS value extracted. The described processing procedure was applied to each muscle signal individually. In summary, two average RMS values from each gait phase for each of the eight muscles were computed for each recorded session.

#### *Kinovea*^®^

The kinematics and spatiotemporal parameters of the 6MWT were analyzed before and after the intervention (indicating the order of not walking after the intervention to all the subjects), and the measurements were performed in synchrony with the sEMG measurements for the first 3 m of the 6MWT. Sagittal plane video recording with reflective markers in the assessed joints [19] was performed. Gait speed was measured during the first 3 m of the 6MWT, and stride length and hip, knee and ankle kinematics during the initial swing (IS) and midstance (MS) phases were measured in the right leg because they are reciprocal phases (Fig. 3). In addition, the step of the first cycle was recorded, expressed as a percentage, and executed within the first 3 m of the 6MWT.

### Statistical analysis

The SPSS (version 23.0) statistical package was used for analysis. Demographic and biometric variables are expressed as the means and standard deviations for quantitative values. The qualitative variables are presented as percentages. MANOVA with Bonferroni adjustment was performed for between-group comparisons. For measures in which a normal distribution could not be assumed post-intervention (according to the Shapiro‒Wilk test), the Kruskal‒Wallis test was applied to each independent variable for comparison between groups. Nonparametric tests (Wilcoxon’s) were applied for related samples for intragroup analysis. The statistical analysis was performed with a 95% confidence interval, and the significant values were those with *p* < 0.05. The effect size was calculated via Cohen’s d.

The difference in the average EMG RMS values for each muscle between the different groups in the pre-intervention recordings was tested independently for the two gait phases by means of a multivariate analysis of variance (MANOVA). Pairwise differences were tested with the Bonferroni post hoc correction in the case of significant main effects. Statistical significance was considered as *p* < 0.05.

The difference in the average EMG RMS values for each muscle between the pre- and post-intervention recordings of the participants in the Vojta group who showed reflex locomotion was analysed independently for the two gait phases considered. For that purpose, the paired-samples t-test was applied after confirming the normality of the populations with the Shapiro‒Wilk test. Statistical significance was considered as *p* < 0.0005 (Bonferroni correction).

## Results

The sample comprised a total of 120 subjects, 54 men (30.4 years ± 10.7) and 66 women (34.2 years ± 12.0) (Table 1). The groups did not differ significantly in age, sex, height or weight.

**Table 1:** Demographic and biometric variables of the study sample for each experimental group and the between-group statistics

Avg. (Std.)/% (#)	Control group *N* = 30	Motomed group *N* = 30	Treadmill group *N* = 30	Vojta group *N* = 30	Statistic
Age (years)	35.1 (11.1)	29.9 (11.5)	35.2 (11.5)	30.3 (11.6)	F(3,116) = 2.010, *p* = 0.116
Sex female	46.6% (14)	46.6% (14)	60.0% (18)	66.6% (20)	χ^2^(3) = 3.636, *p* = 0.304
Height (cm)	171.0 (7.9)	173.6 (7.4)	169.6 (8.3)	171.9 (8.4)	F(3,116) = 1.332, *p* = 0.267
Weight (kg)	74.3 (10.7)	72.9 (8.8)	73.1 (10.7)	71.0 (10.8)	F(3,116) = 0.529, *p* = 0.663

No significant differences in sample baseline characteristics were found in terms of distance covered in the 6MWT, stride length or walking speed in the first 3 m (Table 2). No significant differences were found between the groups in terms of hip, knee, or ankle kinematics at the IS and MS phases on the VoG (t0) (Table 3).

**Table 2:** Average (standard deviation) spatiotemporal values for each group and time point, and the corresponding statistics

		Vojta group	Treadmill group	Motomed group	Control group	Statistics
6 min. Walk (m)	t0	476.4 (57.1)	456.2 (89.8)	491.6 (93.3)	476.3 (71.2)	F(3,116) = 1.009, *p* = 0.392
	t1	9.0 (8.9)	0.0 (0.0)	0.0 (0.0)	0.0 (0.0)	H(3) = 79.066, *p* < 0.001*
Stride length (cm)	t0	31.7 (8.3)	29.6 (5.8)	30.7 (7.2)	30.5 (6.5)	F(3,116) = 0.455, *p* = 0.714
	t1	8.2 (8.4)	0.0 (0.0)	0.0 (0.0)	0.0 (0.0)	H(3) = 61.234, *p* < 0.001*
Time 3 m (s)	t0	4.1 (0.5)	3.9 (0.6)	4.0 (0.4)	4.0 (0.5)	F(3,116) = 0.538, *p* = 0.657
	t1	126.4 (63.2)	0.0 (0.0)	0.0 (0.0)	0.0 (0.0)	H(3) = 87.824, *p* < 0.001*

t0: Pre-intervention (voluntary gait); t1: post-intervention (automatic gait); m, meters; cm, centimeters; s, seconds.

**Table 3:** Average (standard deviation) kinematic values in degrees (°) for each group time point, and gait phase, and the corresponding statistics

		Vojta group	Treadmill group	Motomed group	Control group	Between-group
Initial swing (IS)						
Hip	t0	24.9 (2.6)	26.1 (3.0)	26.0 (2.6)	25.7 (2.8)	F(3,116) = 1.477, *p* = 0.224
	t1	21.5 (8.6)	N/A	N/A	N/A	
	Within-group	Z=-0.267, *p* = 0.789	-	-	-	
Knee	t0	55.1 (3.1)	55.4 (2.8)	56.9 (2.3)	55.6 (3.1)	F(3,116) = 0.558, *p* = 0.644
	t1	53.8 (14.3)	N/A	N/A	N/A	
	Within-group	Z=-0.259, *p* = 0.796	-	-	-	
Ankle	t0	99.2 (4.3)	99.2 (3.5)	98.4 (3.6)	98.3 (3.4)	F(3,116) = 0.376, *p* = 0.770
	t1	108.3 (13.3)	N/A	N/A	N/A	
	Within-group	Z=-1.785, *p* = 0.074	-	-	-	
Midstance (MS)						
Hip	t0	0.2 (0.5)	0.1 (0.4)	0.4 (0.7)	0.3 (0.7)	F(3,116) = 1.213, *p* = 0.308
	t1	1.8 (10.4)	N/A	N/A	N/A	
	Within-group	Z=-1.307, *p* = 0.191	-	-	-	
Knee	t0	5.7 (1.0)	6.1 (1.2)	6.0 (1.1)	5.9 (1.2)	F(3,116) = 2.113, *p* = 0.102
	t1	8.0 (8.3)	N/A	N/A	N/A	
	Within-group	Z=-0.469, *p* = 0.639	-	-	-	
Ankle	t0	0.3 (0.6)	0.2 (0.4)	0.3 (0.6)	0.1 (0.8)	F(3,116) = 0.484, *p* = 0.696
	t1	1.5 (10.8)	N/A	N/A	N/A	
	Within-group	Z=-2.068, *p* = 0.039*	-	-	-	

t0: Pre-intervention (voluntary gait); t1: post-intervention (automatic gait).

However, in the pre-intervention EMG analysis segmented by events (IS and MS), there were significant differences in all muscles at VoG (*p* < 0.001 and *p* < 0.001, respectively), except in the soleus (*p* = 0.63 and *p* = 0.036, respectively) and anterior deltoid (*p* = 0.11 and *p* = 0.49, respectively).

### Effect of interventions on AG

Three categories of results were distinguished: NON-START AG, or gait cycle less than 20% (VG: 6/30: 20%, MG:0/30: 100%, TG: 0/30: 100%, CG: 0/30: 100%); STARTED AG, or gait cycle more than 20% without having concluded it (VG: 5/30: 16.6%, MG: 0/30: 0%, TG: 0/30:0%, CG: 0/30: 0%); and FINISHED AG, or gait cycle more than 20%, concluding at least one cycle (VG: 19/30: 63.3%, MG: 0/30: 0%, TG: 0/30: 0%; CG: 0/30: 0%).

### Intra-group results

Twenty-four subjects executed ≥ 20% of the gait cycle in the AG from the entire sample, all of whom were grouped in the VG. Among these subjects, 19 completed at least one full cycle.

In the comparison between AG and VoG during the 6MWT, significant differences were found in the following variables: distance covered (432.23 m ± 46.32 in VoG against 13.45 m ± 7.34 in AG, *p* < 0.001, Cohen’s d = 0.56); stride length (30.21 cm ± 7.46 in VoG against 13.20 cm ± 6.49 in AG, *p* < 0.001, Cohen’s d = 0.881); and speed in the first 3 m (4.11 s ± 0.52 in VoG against 93.80 s ± 96.12 in AG, *p* < 0.001, Cohen’s d = 0.104). In the kinematic analysis by phase between the VoG and AG, only ankle dorsiflexion in the MS phase was significantly different, with a mean of 0.3° ± 0.6 in the VoG phase versus 1.5° ± 10.8 in the AG phase (*p* = 0.039) (Table 3).

### Inter-group results

Only the VG obtained quantitative and qualitative data that were different from the baseline data (initial position of the 6MWT) in terms of the spatiotemporal and kinematic variables in the 6MWT post-intervention. Thus, in the comparison between groups, significant differences were found in distance covered in the AG (*p* < 0.001, Cohen’s d = 1.52), in cycle phase kinematics evaluated, IS (*p* < 0.001) and MS (*p* < 0.001), in stride length (*p* < 0.0005; Cohen’s d = 1.50) and gait speed in the first 3 m (*p* < 0.001; Cohen’s d = 1.58). There were no differences in these variables between the TG, MG and CG groups (*p* = 1.00) (Table 2).

### EMG results

**Fig. 4:**
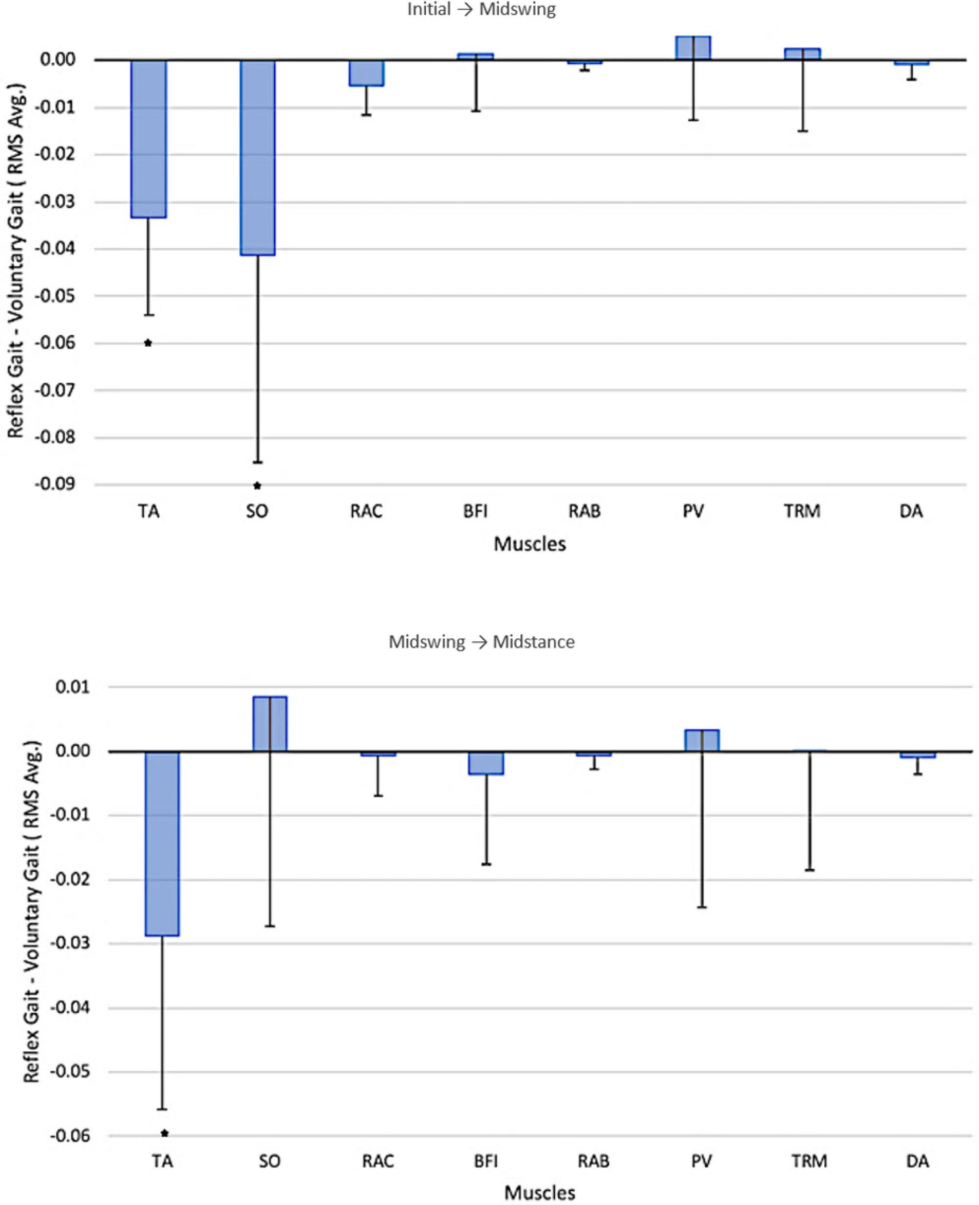
Differences in the average RMS EMG between post-intervention automatic gait and pre-intervention voluntary gait for all muscles considered on the x-axis and for two gait phases (top and bottom). The error bars denote the standard deviations of the differences. *Statistically significant after Bonferroni correction (*p* < 0.001). TA: Tibialis anterior; SO: Soleus; RAC: Rectus femoris; BFI: Biceps femoris; RAB: Rectus abdominis; PV: Paraspinal; TRM: Trapezius; DA: Deltoid

The statistical analyses revealed significant differences, after Bonferroni correction (*p* < 0.001), in the average RMS between the pre- and post-intervention measurements only in the tibialis anterior (TA) muscle in both gait phases and also in the soleus (SO) muscle in the initial gait phase, indicating that a higher degree of muscle contraction occurred before intervention (Table 4; Fig. 4).

**Table 4:** Average EMG RMS (standard deviation) for the pre-intervention voluntary gait and the post-intervention automatic gait, during two separate gait phases, and the statistics of their differences for each muscle considered

EMG RMS Avg. (std.)	Initial → Midswing			Midswing → Midstance		
	Voluntary gait Pre-intervention	Automatic gait Post-intervention	Statistic	Voluntary gait Pre-intervention	Automatic gait Post-intervention	Statistic
Tibialis anterior	0.0538 (0.0207)	0.0205 (0.0106)	t(20)=-7.355 *p* < 0.001 *	0.0646 (0.0310)	0.0353 (0.0152)	t(20)=-4.859 *p* < 0.001 *
Soleus	0.0764 (0.0470)	0.0350 (0.0159)	t(20)=-4.313 *p* < 0.001 *	0.0374 (0.0272)	0.0449 (0.0240)	t(20) = 1.088 *p* = 0.29
Rectus femoris	0.0121 (0.0067)	0.0068 (0.0032)	t(20)=-3.822 *p* = 0.001	0.0103 (0.0052)	0.0094 (0.0055)	t(20)=-0.424 *p* = 0.68
Biceps femoris	0.0135 (0.0081)	0.0148 (0.0110)	t(20) = 0.496 *p* = 0.63	0.0161 (0.0083)	0.0142 (0.0091)	t(20)=-1.157 *p* = 0.26
Rectus abdominis	0.0085 (0.0037)	0.0079 (0.0034)	t(20)=-1.846 *p* = 0.80	0.0092 (0.0044)	0.0085 (0.0047)	t(20)=-1.126 *p* = 0.22
Paraspinal	0.0088 (0.0046)	0.0140 (0.0215)	t(20) = 1.337 *p* = 0.20	0.0119 (0.0067)	0.0153 (0.0307)	t(20) = 0.541 *p* = 0.59
Trapezius	0.0158 (0.0088)	0.0182 (0.0218)	t(20) = 0.612 *p* = 0.55	0.0197 (0.0139)	0.0198 (0.0211)	t(20) = 0.044 *p* = 0.97
Deltoid	0.0053 (0.0031)	0.0045 (0.0019)	t(20)=-1.106 *p* = 0.28	0.0054 (0.0030)	0.0044 (0.0025)	t(20)=-1.788 *p* = 0.09

*Statistical significance after Bonferroni correction.

## Discussion

Considering our findings, a single stimulation session of RLT initiates, reproduces, and maintains the AG pattern in able-bodied persons compared with other interventions, which had no effect on the 6MWT post-intervention. To the best of our knowledge, this is the first RCT that has explored the effects of RLT on AG in able-bodied individuals.

Gait automatisms have not been isolated in able-bodied persons, requiring involvement and dependence on volitional and/or emotional commands for their reproduction. These results could contribute to explaining the results described on gait in children [20, 21] and adults with neurological disorders treated with the RLT [10–12, 22, 23], where variability registered within the VG could be related to different sensitivity profiles to a somatosensory stimulus [7], session frequency [24], and exposure time to stimulus [25, 26], which are considered critical variables in the effects achieved [27].

Our results suggest that the AG pattern was not kinematically ideal, as shown by a foot dorsiflexion deficit at the IS, as confirmed by tibialis anterior sEMG activity. This abnormality could be attributed to the different degrees of sensitivity to reflex activation described by Laufens et al. [28] and to the threshold time necessary to reach the reflex activation variable between subjects [29]. In addition, the great difference in spatial–temporal parameters between VoG and AG is justified by volitional/emotional command absence, which could be mistakenly confused with the immature patterns of toddlers as they begin to walk [30]. Additionally, these findings could be related to the dose and protocol used in the present study. The degree of physical intensity established could be considered low in all interventions, i.e., 3 km/h in the TG, 60 rpm in the MG and nonvoluntary activity requested in the CG and VG. There are no prior studies to compare our findings. However, in the clinical setting, intensity seems to be a differential variable, as it was described by Pavlikova et al. [31] with people with multiple sclerosis, concluding that intensive task-oriented therapy was superior to RLT. Nonetheless, nonrandomization to intervention, nor Expanded Disability Status Scale (EDSS) disability homogenization (higher in the RLT group; *p* = 0.006) and a similar frequency between interventions must be considered in this research. In contrast, similar studies on multiple sclerosis have shown that RLT is superior to other interventions in terms of functional activities [10, 32]. On the other hand, with respect to RLT intensity, adding sessions by training patients in self-management of RLT has also been shown to enhance results [33, 34]. Furthermore, the position of the subject used in our study with the RLT could involve not only specific gait patterns [8], but other studies have also used the same position to activate motor patterns that belong to ideal neurodevelopment [3], such as automatic trunk control [35–37], which is why this position was used in our research.

In terms of sEMG activity, differences in VG pre-intervention in contrast to other groups are related to the higher proportion of women in this group [38]. Post-intervention data support the relationship between the VoG and AG (except for the tibialis anterior), although with higher muscle recruitment rates in the AG. Thus, the AG follows the pattern of cyclical pattern coordination of locomotion in accordance with previous studies [39, 40] and employs sEMG in able-bodied persons and the RLT reflex pattern. sEMG data in our study are compatible with these findings and those described by Bauer et al. in children with cerebral palsy [41]. These data support the existence of neural reciprocity between different crossed locomotion modes (creeping, crawling and walking) because these locomotion modes share cyclical phases of coordination [42] and the same activation of neural nuclei [43].

The data obtained in our study suggest that the modulation of neural circuits that control gait automatisms is a plausible cause of our results. This finding might support the hypothesis of the existence of somatic memory, with a phylogenetic basis, which would relate RLT to neural structures that govern automatic gait processes [44]. The present study could contribute to the development of theoretical-clinical approaches that help to better understand gait neurokinesiological mechanisms. However, although spinal cord reflexes play an important role in gait [45], an independent AG cannot be processed exclusively through reflexes since it requires, at least, the participation of supraspinal structures such as the brainstem, cerebellum, and basal ganglia [46]. In addition, fMRI studies during RLT application seem to support the participation of these subcortical structures and show neuromodulatory effects in areas of the mesencephalic region [47] and in the basal ganglia [48]. Similarly, Martinek et al. [49] demonstrated, using EEG, that the RLT modulates electrical activity in brain areas responsible for planning, regulating, and executing movement. Therefore, future studies using objective assessments and other imaging techniques should be conducted to corroborate our findings in people with neurological disorders.

### Limitations

This study has several limitations. First, it would be necessary to delve into other assessment and imaging systems (such as fMRI) to allow us to track structures involved in the AG and to detect neuromodulation phenomena in neural circuits that we can only infer in our study. Second, the intervention carried out in this research focused on a single session, and its medium-term effects have not been studied. Third, it would be interesting to compare the results between RLT and electromechanical robotic interventions. Fourth, more objective gait measurement instruments, such as photogrammetry systems, that allow human movement analysis in a sensitive way, can be used to explore kinetic and kinematic responses in both lower limbs.

## Conclusions

AG is accessible reflexively and in isolation from other processing systems in able-bodied persons by applying RLT. VG intervention resulted in significant differences in the 6-minute walking test post-intervention, in the distance covered, spatiotemporal and kinematic parameters, and sEMG activity compared with the other intervention groups, which in turn did not present significant intragroup differences or effects on AG processing for any of the variables evaluated in this study. Within the VG, there was variability in the responses, with significant differences between the AG and VoG in terms of ankle kinematics, sEMG activity of the tibialis anterior, stride length, and time to reach the first 3 m of the 6MWT. These results support the idea that RLT has clinical potential and should be explored accordingly in future studies.

## Data Availability

The data generated during this study are not publicly available due to IPR issues, but the preprocessed data that support the findings of this study are available from the corresponding author upon reasonable request.

## Abbreviations

♀: Female
♂: Male
10MWT: 10-meter walk test
1P: First position
6MWT: 6-minute walking test
ANOVA: Analysis of variance
AG: Automatic gait
BF: Biceps femoris
BFI: Biceps femoris
CG: Control group
DA: Anterior deltoid
EDSS: Expanded Disability Status Scale
EEG: Electroencephalography
EMG: Electromyography
EO: External abdominal oblique
fMRI: Functional magnetic resonance imaging
IS: Initial swing phase
MANOVA: Multivariate analysis of variance
MG: Motomed group
MLR: mesencephalic locomotor region
MS: Midstance phase
MT: Mid trapezius
PV: Paraspinal
RAB: Rectus abdominis
RAC: Rectus femoris
RF: Rectus femoris
RLT: Reflex locomotion therapy
RMS: Root mean square
RPM: Revolutions per minute
sEMG: Superficial electromyography
SENIAM: Surface electromyography for the non-invasive assessment of muscles
SD: Standard deviation
SO: Soleus
TA: Tibialis anterior
TG: Treadmil group
TRM: Trapezius
VG: Vojta group
VoG: Voluntary Gait
